# Quantifying Global Tolerance of Biochemical Systems: Design Implications for Moiety-Transfer Cycles

**DOI:** 10.1371/journal.pcbi.1000319

**Published:** 2009-03-20

**Authors:** Pedro M. B. M. Coelho, Armindo Salvador, Michael A. Savageau

**Affiliations:** 1 Biological Chemistry Group, Chemistry Department, University of Coimbra, Coimbra, Portugal; 2 Biomedical Engineering Department, University of California Davis, Davis, California, United States of America; 3 Center for Neurosciences and Cell Biology, University of Coimbra, Coimbra, Portugal; Washington University, United States of America

## Abstract

Robustness of organisms is widely observed although difficult to precisely characterize. Performance can remain nearly constant within some neighborhood of the normal operating regime, leading to homeostasis, but then abruptly break down with pathological consequences beyond this neighborhood. Currently, there is no generic approach to identifying boundaries where local performance deteriorates abruptly, and this has hampered understanding of the molecular basis of biological robustness. Here we introduce a generic approach for characterizing boundaries between operational regimes based on the piecewise power-law representation of the system's components. This conceptual framework allows us to define “global tolerance” as the ratio between the normal value of a parameter and the value at such a boundary. We illustrate the utility of this concept for a class of moiety-transfer cycles, which is a widespread module in biology. Our results show a region of “best” local performance surrounded by “poor” regions; also, selection for improved local performance often pushes the operating values away from regime boundaries, thus increasing global tolerance. These predictions agree with experimental data from the reduced nicotinamide adenine dinucleotide phosphate (NADPH) redox cycle of human erythrocytes.

## Author Summary

The ability of organisms to survive under a multitude of conditions is readily apparent. This robustness in performance is difficult to precisely characterize and quantify. At a biochemical level, it leads to physiological behavior when the parameters of the system remain within some neighborhood of their normal values. However, this behavior can change abruptly, often becoming pathological, as the boundary of the neighborhood is crossed. Currently, there is no generic approach to identifying and characterizing such boundaries. In this paper, we address the problem by introducing a method that involves quantitative concepts for boundaries between regions and “global tolerance”. To illustrate the power of these concepts, we analyzed a large class of biological modules called moiety-transfer cycles and characterized the specific case of the NADPH redox cycle in human erythrocytes, which is involved in conferring resistance to malaria. Our results show that the wild-type system operates well within a region of “best” local performance that is surrounded by “poor” regions.

## Introduction

Robustness, the notion that biological systems must be able to withstand a variety of perturbations is becoming a cornerstone of research in systems biology. Indeed, several approaches have been developed to understand this concept. These approaches tend to focus on the levels of genotype, intermediate network architectures, or phenotypic expression. None actually provides any relation between these levels because the fundamental mappings between levels have not been solved.

At the level of the genotype, there are approaches dealing with neutral or near neutral mutations, which may be considered the result of a genetic code optimized by natural selection. These include nucleotide substitutions that leave the secondary structure of an RNA unchanged [1], that result in a synonymous codon that leaves the protein sequence unchanged, or that lead to the substitution of an aminoacid with similar physical-chemical properties [2]. The fraction of mutations that fall into these classes provides a measure of the organism's “mutational robustness”.

At the level of intermediate network architectures, there are approaches dealing with the number of redundant paths between points in the network. The number of such redundancies provides another measure of robustness. Perhaps the best example of such architectures is provided by networks at the metabolic level [3]. However, these approaches at the level of genotype and network architecture have little to say about any specific biological function.

At the level of specific phenotypic function, the concept of robustness deals with the relationship between the physiological behavior and the underlying parameters of mechanistic models identified or hypothesized. Most approaches at this level have dealt with the local behavior as characterized by small (infinitesimal) changes. Robustness according to these approaches corresponds to parameter insensitivity–linear sensitivities [4], logarithmic sensitivities [5],[6], or second-order sensitivities [7]–[9]. All of these approaches have shown what has been long known from experimental studies, that there is a spectrum of sensitivities with many parameters having very little influence and a smaller number having the major impact.

There are other approaches that attempt to deal with local changes in parameter values analytically, but only in terms of preserving system stability. For systems with a stable steady state, parameter variations that lead to the loss of stability will first violate one of the last two Routh criteria. The magnitudes of these two conditions can be considered a measure of the “distance” from the boundaries of instability. This distance is often referred to as the margin of stability. The margin in the case of the penultimate condition is the more difficult to evaluate; it involves both kinetic order and rate constant parameters [10]–[12]. The margin in the case of the last Routh criterion is determined more simply by the determinant of the matrix of kinetic orders for the dependent variables [10],[13], alternatively by a method based on singular value decomposition of this matrix [14]. For many systems both conditions are critical and must be evaluated. However, these local approaches have little to say about a system's response to larger changes in parameter values.

One approach to deal with large changes in parameter values involves random sampling of values to obtain an estimate for the volume of parameter space corresponding to physiological behavior [15], although volume alone is not a sufficient measure. The shape of the volume is critical, as pointed out by Morohashi *et al.* [16]. Sengupta *et al.* [17] and Chaves *et al.* [18] have proposed a measure of robustness, based on a random walk in parameter space, that reflects the shape of the robust region. These methods are limited by the computational expense of dense sampling and random walks in high-dimensional parameter spaces.

All of the existing methods have advantages as well as significant limitations. Thus, there is need of a generic approach for dealing with robustness to large changes in parameter values and identifying a variety of qualitatively distinct phenotypes, including but not limited to loss of stability. In this paper, we introduce such a method and illustrate its use in the context of a specific class of biochemical systems, moiety-transfer cycles. In such systems, the variables and parameters, which define its structure, must remain within a neighborhood of their nominal values so as to produce a physiological phenotype. When this neighborhood is exceeded the system exhibits a pathological phenotype.

Our generic approach involves the precise characterization of boundaries between phenotypically distinct regimes and defines “global tolerance” as the ratio (or its reciprocal, depending on which is greater) between the normal value of a parameter and the value at such a boundary where there is an abrupt change in system performance. Thus, systems whose performance remains nearly constant for large deviations from the normal operating point are considered to be “globally tolerant”. This is in contrast to the conventional notion of “local robustness”, defined by small values for the system's parameter sensitivities [5], which results in important aspects of system performance remaining almost constant near the normal operating point. As biochemical parameters might be subject to considerable variation, a small global tolerance might be disadvantageous even if system performance is locally robust.

The notion that large global tolerances may evolve as “safety factors” against fluctuations in parameter values and/or in the loads placed by the environment has been proposed as a possible explanation for large mismatches found between actual biological capacities and apparent physiological needs [19]–[22]. For example, the measured capacity (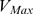 value) of hexokinase exceeds the physiological flux in the cardiac muscle of exercising rainbow trout by over three orders of magnitude [21]. More recent studies [23],[24] of concrete systems suggest that large tolerances of pathway fluxes to changes in the activity of the participating enzymes are the side-effect of fulfilling local performance criteria. However, we can envision a situation in which effective local performance will not necessarily lead to large tolerances, and therefore the possibility of performance breakdown due to normal variation in parameter values becomes a major consideration mediating natural selection. A similar point is highlighted by Morohashi M, *et al.* [11], showing that various aspects of the design for a biochemical oscillator can be rationalized as attending to a requirement for both good local performance and large global tolerance. Therefore, local robustness and global tolerance are both important aspects for the evolutionary design of biochemical systems.

**Figure 1:**
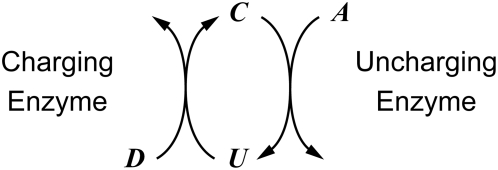
Schematic representation of a moiety-transfer cycle. The symbols 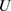 and 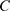 represent the moiety-uncharged and moiety-charged carrier, respectively, and 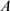 and 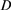 represent the moiety-acceptor and moiety-donor metabolites, respectively. The sum 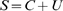 is conserved under the conditions of interest here.

In illustrating our generic approach, we also will address the question: does design for robust local performance necessarily improve global tolerance? In moiety-transfer cycles, a moiety is transferred from a moiety-donor metabolite (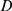) to an acceptor metabolite (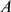) by way of a charged carrier (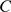) (Figure 1). For our example, and under the conditions of interest, we will assume that the sum (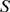) of the charged carrier (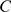) and the uncharged carrier (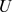) is held constant. This form of coupling between reactions is very prevalent in metabolism. Indeed, of all the enzyme-catalyzed reactions in the reconstructed metabolic networks of *Escherichia coli* [25] and *Saccharomyces cerevisiae* [26], 836 (75%) in the former organism and 561 (67%) in the latter participate in moiety-transfer cycles. These calculations exclude cycles involving the ubiquitous metabolites H_2_O and H^+^, and pairs of forward-reverse reactions. Redundant reactions catalyzed by distinct (iso)enzymes were counted as a single reaction.

The large majority of these cycles mediate the transfer of moieties from catabolic (i.e., nutrient-disassembling and energy-producing) to anabolic (biosynthetic) processes. In this context, they act as “moiety-supply” units, analogous to power-supply units in electric circuits: they must reliably supply a given moiety at the required rate (analogous to current intensity) while keeping the concentration of the charged carrier (analogous to electric potential) fairly constant. Here we address moiety-transfer cycles that play this specific role. Henceforth, when we use the term “moiety-transfer cycles” it should be understood that we are referring specifically to the class of moiety-transfer cycles that act as “moiety-supply” units. We also compare our analytical results to existing experimental results for the NADPH redox cycle of human erythrocytes.

## Methods

### Model Formulation

We will assume that each enzyme involved in a moiety-transfer cycle (Figure 1) has two substrates and that the reactions are irreversible. For our particular example, we will use Eqn (1), which is valid for a wide range of two-substrate enzymatic mechanisms (random-order equilibrium, compulsory-order, Theorell-Chance and ping-pong mechanisms) [27]:
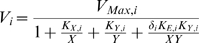
(1)where: 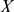 is the concentration of substrate 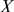; 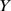 is the concentration of substrate 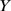; 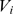 is the rate of catalysis by enzyme 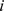; 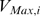 is the maximum rate of catalysis by enzyme 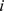; 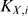 is the Michaelis constant of enzyme 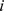 with respect to substrate 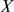; 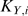 is the Michaelis constant of enzyme 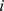 with respect to substrate 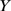; 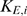 is the equilibrium dissociation constant for the enzyme-substrate complex 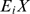; 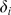 is 1 if the enzyme follows a random-order equilibrium or a compulsory-order mechanism in which 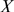 binds first and 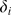 is 0 if the enzyme follows a ping-pong mechanism.

For purposes of illustration, we will assume that the charging enzyme follows a compulsory order mechanism in which 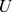 binds first to the enzyme (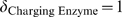) and the uncharging enzyme follows a ping-pong mechanism (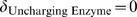). For simplicity, and without ambiguity since we are only considering two different enzymes, we are going to discontinue using the subscript referring to the enzyme. Hence the terminology that we are going to use throughout the text is as follows (see Figure 1):
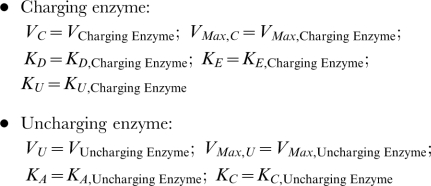



### Strategy for Analysis

The investigation of tolerance requires a mathematical framework that is able to address the effects of large perturbations while avoiding the mathematical complexities of unstructured nonlinear systems. The strategy for our analysis involves (i) decomposition of the system's design space into unique regions with boundaries precisely defined by the “breakpoints” in the piecewise power-law representation, (ii) determination of the system behavior in each region, (iii) evaluation of system behavior according to a set of quantitative criteria based on the function of the system, and (iv) determination of the global tolerance to changes in the values for the parameters and concentrations of the system.

### Piecewise Power-Law Representation

**Figure 2:**
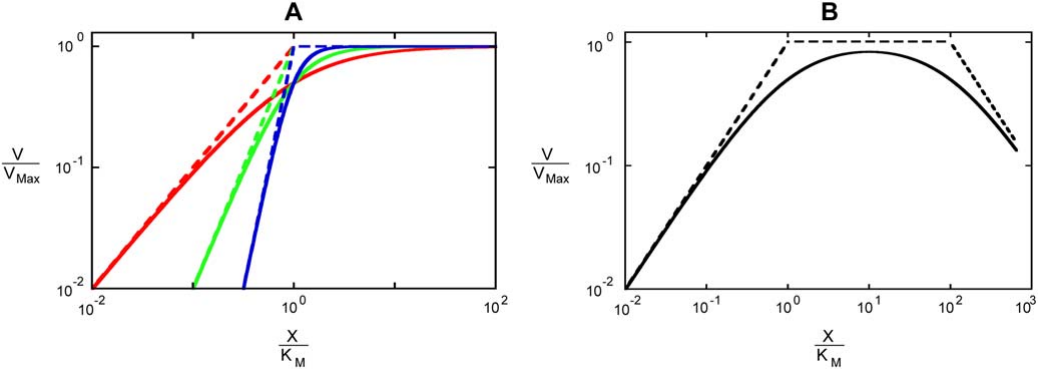
Piecewise Power-Law (dashed line) and Rational-Function (solid line) representations of reaction rate (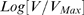) as a function of substrate concentration (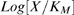). (A) The Hill rate law given by 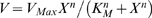. The color indicates the Hill coefficient: (red) n = 1 (Michaelis-Menten); (green) n = 2; (blue) n = 4. (B) The rate law for substrate inhibition given by 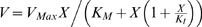. For our particular example, the ratio 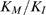 is 10^−2^.

Our approach is based on the idea that performance differs when there is a change in the dominant flux or concentration terms. For instance (Figure 2A), for enzymes that obey the Hill function, the characteristic concentration—typified by the 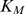
—marks the breakpoint between two regimes in logarithmic space. One is characterized by most of the enzyme being in the free form (slope equal to the Hill coefficient) and the other by most of the enzyme being bound to the substrate (slope equal to zero). More complicated enzyme mechanisms, will involve more than one breakpoint. For instance, some enzymes exhibit substrate inhibition at elevated substrate concentrations (Figure 2B). For these enzymes, there will be three regimes separated by two breakpoints. At substrate concentrations much below the 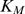, most of the enzyme is in the free form (slope equal to one); at intermediate concentrations, above the 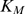 and below the 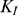, the enzyme is mostly bound by a single molecule of substrate (slope equal to zero); at substrate concentrations much above the 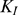, the enzyme is mostly bound in an abortive or dead end complex between the substrate and one or several enzyme forms (slope equal to −1).

The essential feature of a system, and that any mathematical framework for the analysis of tolerance has to capture, is thus the breakpoints between regimes. These ideas lead us to estimate tolerances within the framework of the piecewise power-law representation of enzyme kinetics, which is one of the four different representations within the power-law formalism of Biochemical Systems Theory [28]. This representation retains the mathematical tractability of the local power-law representation [5], which provides a characterization of the system in terms of logarithmic gains, robustness (as measured by parameter sensitivities) and local stability, while extending the range of application to global considerations.

Formulation of our piecewise power-law representation is analogous to the classical method of Bode [29] and involves three steps ([10], pp 335–341):

1. expressing the kinetic rate laws for the enzymes in a factored form (here normalized) that allows us to identify the “poles” (values of the dependent variable that would cause the rate law to approach infinity) and “zeros” (values of the dependent variable that would cause the rate law to approach zero):
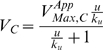
(2)and
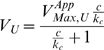
(3)where
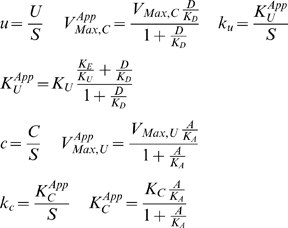
The simple Michaelis-Menten rate law is already in this form, but more complex rate laws will require this factoring step ([10], pp 335–341).
2. normalization of both kinetic rate laws by the apparent 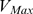 of the charging reaction (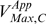)
3. representing each normalized rate law by its asymptotes in Log Space.

Using this method, we derive the piecewise power-law representation:
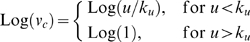
(4)and
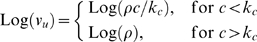
(5)

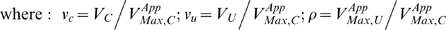
Although the asymptotes in this example are straight lines in both Cartesian and Logarithmic coordinates, this is not the general case. In the general case, the asymptotes are straight lines only in the Logarithmic coordinates.

**Figure 3:**
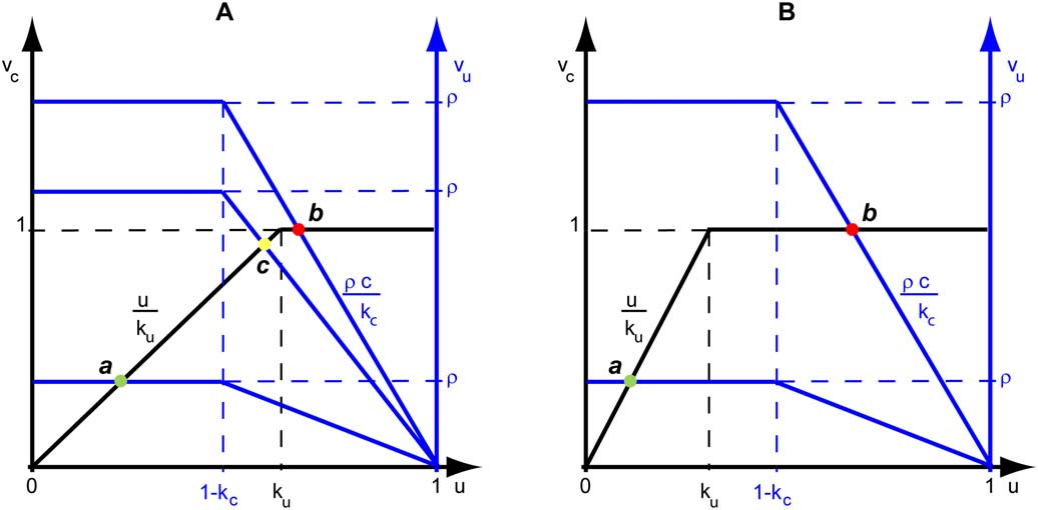
Piecewise power-law representation of normalized rate vs. normalized concentration (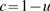): (A) 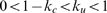; (B) 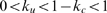. Systemic regimes are colored and labeled as in Figure 4.

Under the condition 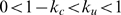 (Figure 3A) there are three different regimes each with a different steady state. For very small values of 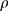, the steady state in Systemic Regime ***a*** is valid. In this steady state, the charging enzyme operates within its linear region and the uncharging enzyme operates on its plateau. As 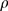 increases, there is a transition to the steady state in Systemic Regime ***c***, in which both enzymes operate within their linear regions. Finally, as 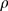 increases even further, there is a transition to the steady state in Systemic Regime ***b***, in which the charging enzyme operates on its plateau and the uncharging enzyme functions within its linear region.

Under the condition 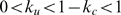 (Figure 3B) there are two different regimes each with a different steady state. For values of 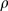 less than one, the steady state in Systemic Regime ***a*** is valid; when 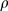 equals one the system experiences a discontinuity and transitions to the steady state in Systemic Regime ***b*** for values of 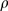 greater than one.

**Figure 4:**
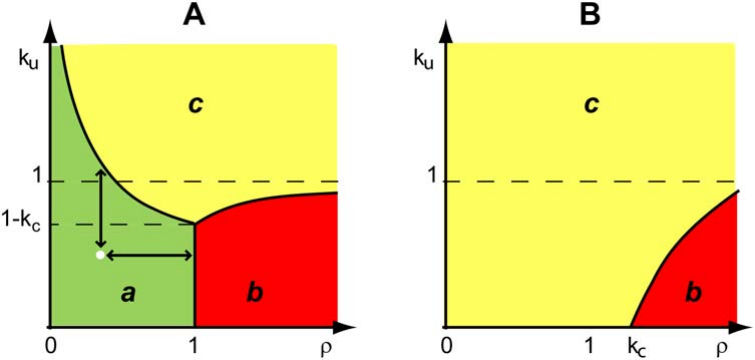
Design space of the moiety-transfer cycle. Three distinct operating regimes labeled *a*, *b* and *c* are depicted for the model in Figure 1: (A) 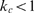 and (B) 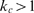. See text for discussion.

Through the analysis of these cases, and of the remaining ones (see Text S1), we are able to determine the design space available to the moiety-transfer cycle (see Figure 4).

Each systemic regime is given by a specific and readily solvable steady-state equation for the dependent variable, and applies only to a particular region of the design space (Table 1). Given this partitioning of the design space into distinct regions, one can define global tolerance as the ratio between the value of a parameter at the operating point (white point in Figure 4A) and the value of that same parameter at the boundary to the next neighboring region (black double headed arrows in Figure 4A).

**Table 1:** Steady-state solution in each of the systemic regimes.

*Regime*	*Steady-State Concentration * 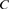
***a***	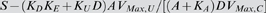
***b***	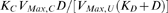
***c***	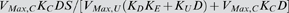

### Determination of System Behavior within Each Regime

The system representation within each regime is a simple but nonlinear S-system for which determination of local behavior, after appropriate transformation, reduces to conventional linear analysis [10]. Thus, the local behavior is completely determined and readily characterized by the evaluation of the following quantitative indices.

*Logarithmic gains* in concentration (*e.g.*, the charged moiety 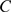) or flux (*e.g.*, the rate of charged-moiety supply 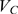) in response to change in value for an independent variable (*e.g.*, the concentration of the moiety-acceptor 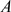) are defined by the relative derivative of the explicit steady-state solution. For example,
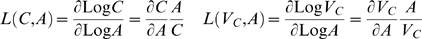
(6)


*Parameter sensitivities* of such state variables in response to change in the value for one of the parameters that define the structure of the system (*e.g.*, Michaelis constants or maximal velocities) are defined by the relative derivative of the explicit steady-state solution. For example,
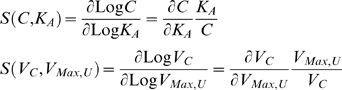
(7)


*Response time* is given by the inverse of the eigenvalue, which is determined by analytical integration of the differential equation that applies for each systemic regime.

### Criteria for the Proper Operation of a Moiety-Transfer Cycle

What criteria must a moiety-transfer cycle fulfill in order to be considered a good one? This is a question that only now is being posed by biologists. However, this question is analogous to one that engineers have long had to deal with, and the lessons they have learned can now be used to further our understanding of how biological systems are designed through natural selection.

The performance of the moiety-transfer cycle, which is analogous to that of the power supply in an electrical circuit, can be evaluated in each systemic regime according to the following quantitative criteria:

The concentration of charged carrier 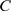 (analogous to the voltage of the power supply) should be well buffered against:

Criterion 1: fluctuations in the values of the kinetic parameters of the enzymes and of the independent variable 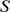 (A power supply should not be sensitive to, for instance, changes in the properties of its internal components due to temperature variations);
Criterion 2: changes in the concentration of moiety-acceptor 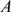 (The voltage of a good power supply should not drop significantly when there is an increase in demand for more current);
Criterion 3: changes in the concentration of moiety-donor 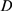 (The voltage of a power supply should not drop significantly when there is a decrease in the line voltage).

The supply of charged carrier 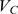 (analogous to the electrical current) should

Criterion 4: be responsive to changes in the concentration of moiety-acceptor 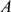 (A good power supply should be able to supply more current when it is needed).

The sensitivity of the supply of charged carrier 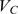 to changes in the concentration of moiety-acceptor 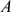 should

Criterion 5: be well buffered against fluctuations in the values of the kinetic parameters of the enzymes and independent variables (When you are demanding more current from the power supply, you do not expect the output to depend on, for instance, temperature)

The response time should

Criterion 6: be fast (when demanding more current from the power supply, you do not expect a prolonged delay in the response), and
Criterion 7: well buffered against fluctuation in the values of the kinetic parameters and independent variables (The response time of the power supply should be reproducible in spite of such fluctuations).

## Results

The local performance in the three systemic regimes is determined by the above methods and evaluated according to the criteria defined in the previous section. Our aim is to ascertain which of the systemic regimes is better suited for effective performance of the moiety-transfer cycle as a moiety-supply unit. Note that if this same cycle were to fulfill a different role in the cell, then we would have to define different criteria and, hence, the results could be different. For instance, Golbdeter and Koshland [30] have studied a different type of moiety-conserved cycle that exhibits ultra-sensitivity and switch-like behavior.

Optimum local performance of systems with respect to each criterion and within each regime corresponds to the minimum value possible for the criterion (Optimum Value).

### Analysis of Local Performance

In Table 2, we summarize the results from the analysis of local performance in Systemic Regime ***a***. (Details of these results are presented in Text S2) It is apparent from these results that the performance in Systemic Regime ***a*** fulfills all of the criteria defined above. Furthermore, if Condition 1, 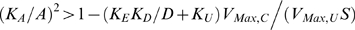, is valid, the optimization of criteria *1* through *6* follows the same strategy: 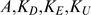 and 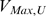 should decrease while, 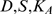 and 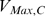 should increase. Note that there is one apparent conflict between optimizing Criterion 7 along with the previous criteria. In order to optimize criteria 1, 2 and 6, 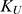 should tend to low values, whereas to optimize performance according to Criterion 7, 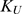 should tend to high values. This apparent conflict can be readily resolved with appropriate values for 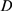, 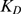 or 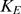 (for which there are no trade-offs).

**Table 2:** Evaluation of the Local Performance in Systemic Regime ***a***.

*Criterion*	*Optimum Value*	*Parameters/Variables that Correlate Negatively*	*Parameters/Variables that Correlate Positively*
1	1	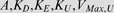	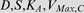
2	0	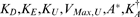	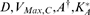
3	0	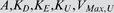	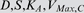
4	1	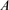	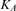
5	0	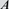	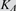
6	0	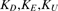	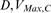
7	2	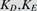	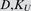

^*^ Condition 1(see text) true.

^†^ Condition 1 false.

Contrary to the results for Systemic Regime ***a***, the performance in Systemic Regimes ***b*** and ***c*** cannot fulfill criteria *4* and *5* because there is no response to changes in moiety-acceptor 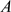 (detailed results in Text S2). In addition, even though the performance in Systemic Regimes ***b*** and ***c*** can have a fast response time (Criterion 6), it will not be with respect to changes in 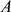. Therefore, the importance of this responsiveness becomes questionable. Finally, the optimum value of Criterion 1 in Systemic Regime ***c*** is 1, whereas that in Systemic Regime ***b*** is 3. Since Systemic Regimes ***b*** and ***c*** share the same optimum values for the remaining criteria, we conclude that overall local performance in Systemic Regime ***c*** is better than that in Systemic Regime ***b***.

### Identifying the Region of Best Local Performance

From the analysis of local performance, it is clear that the only systems that can fulfill all criteria and do it efficiently operate in Systemic Regime ***a***. Although systems that operate in systemic regimes ***b*** and ***c*** can fulfill some of the performance criteria, they fail in that their supply of charged carrier, 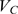, does not respond to changes in the concentration of moiety-acceptor 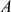. In analogy to electrical circuits, they resemble a power supply that will not provide additional current when there is an increased demand by the rest of the circuit. Hence, this is a poor design for a power supply unit.

If there had been no regime capable of simultaneously fulfilling all the performance criteria then one would have to evaluate the relative impact on fitness of the failure to satisfy a specific criterion. Regimes that violate performance criteria with a weak effect on fitness would clearly be preferable to those that violate more important performance criteria. If the results showed that all regimes violated important performance criteria, then one may attribute this to an inappropriate model or to incomplete/inaccurate knowledge about the function of the system under analysis.

In summary, we predict that in nature, under basal conditions, a moiety-transfer cycle should operate in Systemic Regime ***a***. Moreover, natural selection should maintain the operating point far from the boundaries to the other regimes for the following two reasons. First, the circuit's local performance improves as the operating point moves away from the boundaries. Second, even where the intra-regime gradient in local performance is modest, excursions into neighboring regimes of poor performance are less likely when the operating point is farthest from the boundaries.

### Analysis of Global Tolerance

Systemic Regime ***a*** holds in the region of design space (Figure 4) defined by the following inequalities:
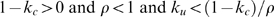
Systems represented within these boundaries exhibit the best local performance and thus these boundaries provide the basis for a natural definition of global tolerance. Namely,

*Global tolerance is given by the ratio (or its reciprocal, depending on which is greater) of the value for each parameter or independent variable at the normal operating point relative to its value at the boundary of the region.*

By the use of this definition it is possible to determine analytically the global tolerance to change for each kinetic parameter and independent variable of the system operating in Systemic Regime ***a***. In general, each parameter or independent variable can have a global tolerance with respect to its lower value as well as its upper value. These tolerance values will be denoted “[T_low_,T_high_]”; since one of these is often infinite, we also will use the notation “[T_low_” or “T_high_]” with the other infinite tolerance implied.

There are two different boundaries for Systemic Regime ***a***, 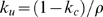 and 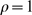, so we present the tolerance expressions with respect to each in Text S3. When considering each kinetic parameter and independent variable individually, its *critical tolerance* will be given by the lowest of its tolerance values given in Text S3. Numerical values for these tolerances are given for a specific system in the following section.

### NADPH Redox Cycle in Human Erythrocytes

**Figure 5:**
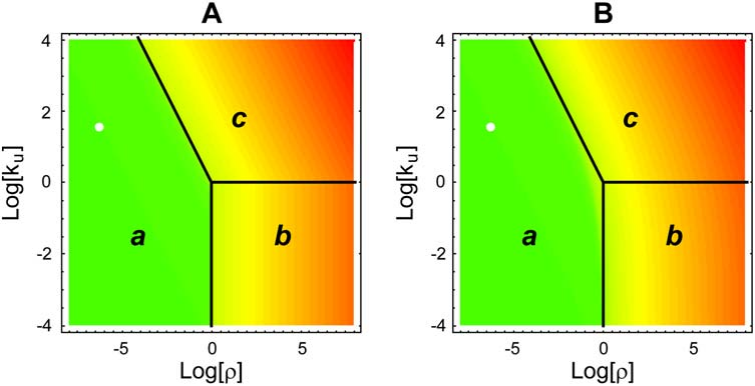
Design space depicting the steady-state solution of the NADPH redox cycle in human erythrocytes: (A) piecewise power-law representation and (B) Michaelis-Menten representation. The color indicates the logarithm of the normalized steady-state concentration of moiety-charged carrier, 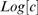: (green) High to (red) Low. The white point in the figure represents the normal operating point of the cycle. The three Systemic Regions are denoted *a*, *b*, and *c*. The boundaries between regions are determined for the piecewise power-law representation and then superimposed on both panels. The Log-Log coordinates provide a more convenient representation of the design space that was shown with Cartesian coordinates in Figure 4A.

We have selected this moiety-transfer cycle to provide a numerical illustration of our results because the kinetic parameters of the enzymes and concentrations of the metabolites for this system have been well characterized experimentally [31]–[34] in view of this cycle's importance in malaria [35]. These values, which are in Text S4, lead to the design space in Figure 5 depicting the steady-state concentration in the z-direction with a heat map. The physiological operating point for this system is found in Systemic Region ***a***, as expected. The design space depicting the steady-state flux has a similar appearance (data not shown).

The local behavior of this system can be evaluated according to the seven criteria described earlier. In this case we have the numerical values for the various parameters and, thus, we can calculate the numerical values for the criteria and compare their values to the optimum values. As can be seen from the resulting data summarized in Table 3, natural selection results in a design that has nearly optimal local performance according to the seven criteria.

**Table 3:** Evaluation of local performance for the NADPH redox cycle in human erythrocytes.

*Criterion*	*Quantitative Value for the Normal Operating Point*	*Optimum Value*
1	1.048	1
2	0.009	0
3	0.003	0
4	1.002	1
5	0.002	0
6	0.198	0
7	2.606	2

Given the numerical values that characterize the operating point for this system, and the boundaries surrounding Systemic Region ***a***, we are able to determine the numerical value of global tolerance for each of the kinetic parameters and independent concentration variables. The values, summarized in Table 4, are tolerances involving movement from Systemic Region ***a*** into Systemic Region ***c***. They range from the smallest tolerance of 59 fold to the largest of 362 fold. The smallest values are associated with 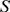, 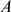, and 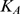, whereas the largest are associated with 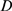, 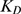, and 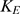.

**Table 4:** Values for tolerances of the NADPH redox cycle in human erythrocytes.

*Variable or Parameter*	*Tolerance*
[G6P] * ( 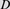 )	[362
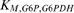 † ( 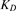 )	362]
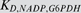 ‡ ( 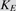 )	362]
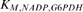 ( 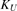 )	158]
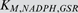 § ( 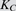 )	126]
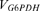 ( 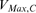 )	[110
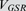 ( 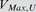 )	110]
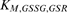 ¶ ( 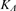 )	[69
[GSSG] ( 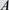 )	69]
[NADP+NADPH] ( 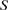 )	[59

*: G6P: Glucose 6-phosphate.

**†:** G6PDH: Glucose-6-phosphate dehydrogenase.

**‡:** NADP: Oxidized nicotinamide adenine dinucleotide phosphate.

**§:** GSR: Glutathione reductase.

**¶:** GSSG: Oxidized glutathione.

It should be emphasized that no change in the value of any *single* parameter or concentration is capable of moving the operating point of the system from Systemic Region ***a*** into Systemic Region ***b***. In this sense, the largest tolerances (essentially infinite) are associated with the boundary between systemic regions ***a*** and ***b***.

## Discussion

The organization of biochemical systems has traditionally been viewed as adhering to few general rules. Should it be real, this perceived lack of generally applicable organizing principles would reduce molecular biology to an accumulation of disparate facts with limited predictive value. However, research in molecular systems biology is revealing a number of design principles that associate function with design. For example, such design principles have been found in metabolic pathways [36]–[40], signal transduction cascades [41]–[45], mode of gene control [28], [46]–[49] and coupling of gene circuits [50]–[54]. This research provides an understanding of why some designs are highly prevalent in biochemical systems while other feasible designs are rare. It also prompts predictive inferences of (i) what interactions among biochemical components should occur given the function of a network, or (ii) what is the likely function of a network given its component interactions.

A high priority in the research program of biochemical systems theory is the characterization of design principles for the most common constituents of biochemical systems such as elementary gene circuits and simple metabolic networks. As noted in the Introduction, moiety-transfer cycles are among the most common functional units in metabolic networks. Hence, the material presented in this paper serves not only to introduce an important analytical framework within which to quantitatively characterize the design of biochemical systems, but also to provide insight regarding the design principles that govern one of the most common functional units in metabolic networks.

It must be emphasized that the piecewise power-law representation described in this paper is not an arbitrary fit to the kinetic rate laws. It is not simply a convenient curve-fitting exercise that attempts to minimize the error in the representation by using a sufficiently large number of arbitrary pieces. The number of pieces, their slopes and the location of the breakpoints are all uniquely determined by the rational function in conventional Bode-type analysis ([10], pp 335–341). Moreover, this representation is rigorously justified for the rational functions known to characterize the traditional rate laws of biochemical kinetics [55]. Thus, the method is highly constrained by the model and it produces a unique representation. The class of models can be quite general; for example, it includes generalized mass action models of chemical kinetics and rational function models of biochemical kinetics. Regardless of how one obtains a given model (detailed kinetic analysis, an empirical fit to a model using limited data or a hypothetical model based on general considerations), as long as it falls within this very general class of functions then our approach can be applied.

Differences between the steady-state solutions of the rational function and piecewise representations are greatest around the breakpoints, as is evident from Figure 5. The lack of accuracy at these points may be considered a disadvantage of the piecewise power-law representation. Nevertheless, the piecewise power-law representation suggests the formulation of the design space, provides precise boundaries between regions, and gives a method for defining global tolerances in a quantitative manner. These are all major advantages that would be hard to derive directly from the rational-function representation. Thus, it must be emphasized that in our example the formulation of the design space and the boundaries were first derived from the piecewise representation (depicted in Figure 5A) and then used to display the results from the rational-function representation (depicted in Figure 5B).

The system design space that is defined by our approach provides an important framework to characterize the behavior of the system. Within each region, system behavior is readily solved, often analytically, as for the cases analyzed in this paper. The results presented in this paper can be generalized to other moiety-transfer cycles, as will be documented in a subsequent publication (Coelho *et al.*, manuscript in preparation).

The system design space also provides an important framework to represent and compare wild-type and mutant variants of these systems. The kinetic parameters of the systems can be measured and the resulting values plotted within the common design space. An example is provided in Figure 5 by making use of the data for the wild-type NADPH redox cycle in human erythrocytes [31]–[34].

The location of the operating point for mutants (where such mutants and their kinetic data are available), in relation to that for the wild type and in relation to the boundaries between good and poor regions, will provide a method to quantitatively characterize the physiological significance of mutant phenotypes.

There is a general theorem indicating that the robustness of feedback control systems is a conserved quantity, and thus increasing the robustness in one operating regime must cause it to decrease in another [56]. This suggests that trade-offs are inevitable in the design of a system. It is not yet clear how our results might be governed by this theorem. The differences may reside in the global dynamics of the system, since our analysis focuses on the steady-state behavior and only considers dynamics in the local sense.

As we have seen, an important consideration affecting the location of the operating point for the wild type relative to regime boundaries is the interplay between global tolerance and local performance. Selection for improved local performance often pushes the operating point away from regime boundaries, thus increasing global tolerance. But in some cases modifying the value of a parameter in the direction that improves local performance may bring the operating point closer to regime boundaries, thus decreasing global tolerance.

Our analysis identified two cases of potential trade-offs between specific criteria for local performance and global tolerance. Namely, increasing 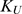 improves the buffering of the response time against fluctuations in the values of parameters and independent variables, but decreases global tolerances with respect to changes in the values of most parameters. Likewise, decreasing 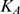 can in some conditions improve buffering against changes in the concentration of moiety-acceptor 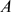, but it can decrease global tolerances with respect to changes in the values of most parameters.

However, because these same changes in 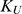 or 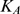 would also worsen several other important aspects of local performance they do not entail a real trade-off between overall local performance and global tolerances. Furthermore, none of the trade-offs mentioned above prevent the simultaneous improvement of both local performance and global tolerance by suitably changing the value of a second parameter. Therefore, the simple design of moiety-transfer cycles that we addressed here does not have any irresolvable trade-offs between global tolerance and local performance for the set of performance criteria we considered. This is a desirable property that facilitates the evolutionary adaptation of the cycle to changing environmental demands.

## Supporting Information

Text S1 Steady-state solutions
(0.48 MB DOC)

Text S2 Analysis of local performance
(0.24 MB DOC)

Text S3 Tolerance expressions
(0.72 MB DOC)

Text S4 NADPH redox cycle in human erythrocytes
(0.07 MB DOC)
