# Supplementary material for: Quantifying Global Tolerance of Biochemical Systems: Design Implications for Moiety-Transfer Cycles
Source: PLoS Comput Biol. 2009 Mar 20;5(3):e1000319. doi: 10.1371/journal.pcbi.1000319 (PMC2650413; doi:10.1371/journal.pcbi.1000319)
Supplement: Text S1 — Steady-state solutions (0.48 MB DOC) [file pcbi.1000319.s001.doc]

**Supporting Text S1**

## *Steady-State Solutions*

Based on the piecewise representation of the rate laws in Log space (Eqns. 4 and 5 of the main text), we recognize that the moiety-transfer cycle can operate under three different meaningful steady-state regimes and one unrealistic case with $u>k_{u}, c>k_{c}, \rho=1$ (This case represents the system on a “knife edge”. A small change in an independent variable or parameter, and the system reverts to one of the three meaningful steady-state regimes.). The particular steady-state regime that is valid depends on $\rho$, $k_{c}$ and $k_{u}$.

Under the condition $k_{u}>1$ and $k_{c}<1$ (Figure S1**A**) there are two different regimes each with a different steady state. For very small values of $\rho$, the steady state in Systemic Regime ***a*** is valid. In this steady state, the charging enzyme operates within its linear region and the uncharging enzyme operates on its plateau. As $\rho$ increases, there is a transition to the steady state in Systemic Regime ***c***, in which both enzymes operate within their linear regions.

Under the condition $k_{u}<1$ and $k_{c}>1$ (Figure S1**B**) there are two different regimes each with a different steady state. For small values of $\rho$, the steady state in Systemic Regime ***c*** is valid. As $\rho$ increases, there is a transition to steady-state Regime ***b***, in which the charging enzyme operates on its plateau and the uncharging enzyme functions within its linear region.

Under the condition $k_{u}>1$ and $k_{c}>1$ (Figure S1**C**), only the steady state in Systemic Regime ***c*** is valid.


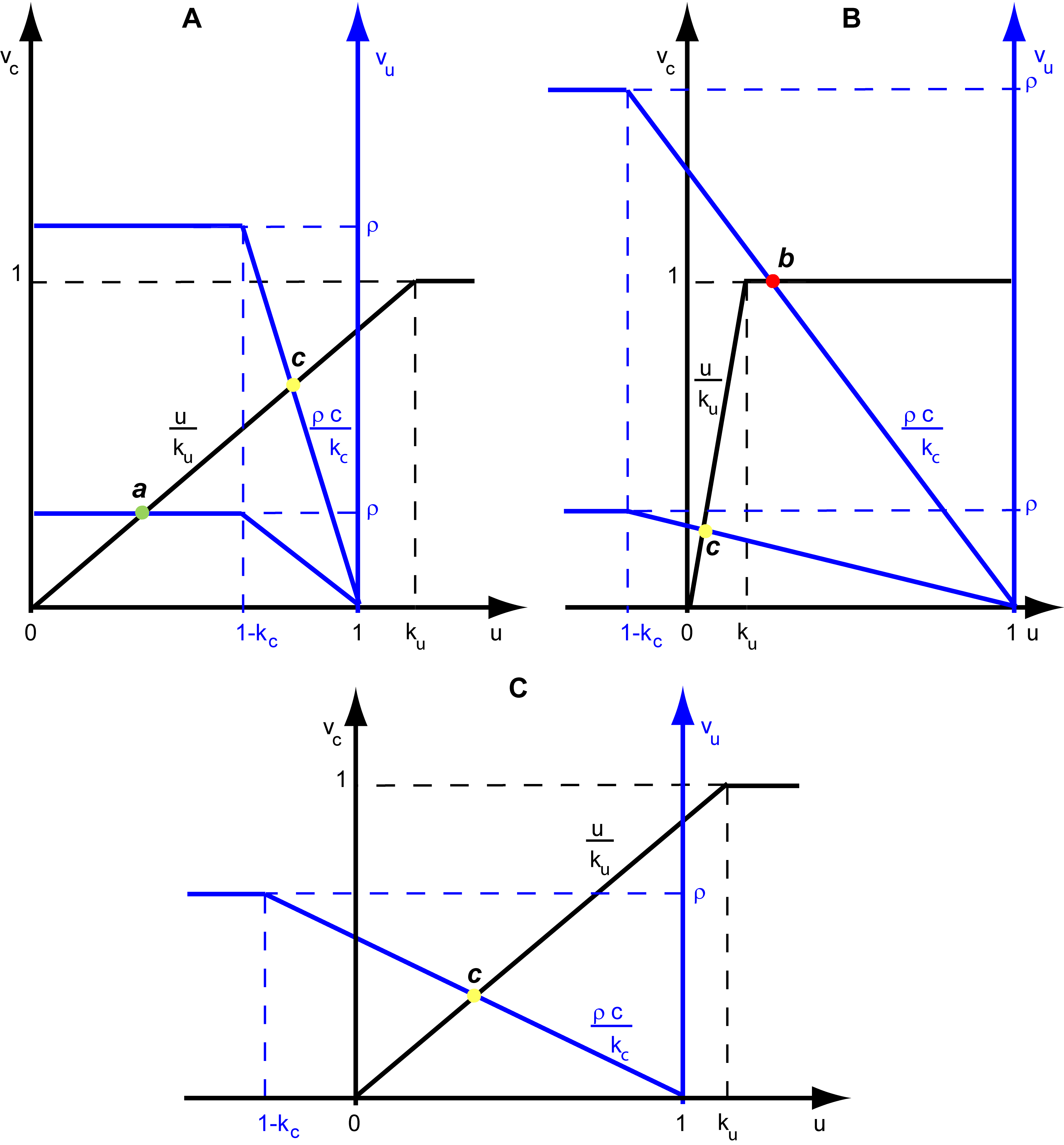


**Figure S1.** Piecewise power-law representation of normalized rate vs. normalized concentration ($c=1-u$): Panel **A**, $k_{u}>1$ and $k_{c}<1$; Panel **B**, $k_{u}<1$ and $k_{c}>1$; Panel **C**, $k_{u}>1$ and $k_{c}>1$. Systemic regimes are colored and labeled as in Figure 4 of the main text.

Through the analysis of these cases, and of the ones discussed in the main text (see Figure 3 of the main text), we are able to determine the design space available to the moiety-transfer cycle (see Figure 4 of the main text).
