# Supplementary material for: Quantifying Global Tolerance of Biochemical Systems: Design Implications for Moiety-Transfer Cycles
Source: PLoS Comput Biol. 2009 Mar 20;5(3):e1000319. doi: 10.1371/journal.pcbi.1000319 (PMC2650413; doi:10.1371/journal.pcbi.1000319)
Supplement: Text S2 — Analysis of local performance (0.24 MB DOC) [file pcbi.1000319.s002.doc]

**Supporting Text S2**

## *Analysis of Local Performance*

## *Systemic Regime a*

In Table S1, we show, for each criterion, the analytical expression that applies in Systemic Regime ***a***. Since optimum local performance with respect to each criterion corresponds to the minimum possible value, we are able to differentiate each analytical expression and determine whether a parameter/variable should decrease or increase to improve performance.

As an illustration, we will consider Criterion *2*. In order to optimize performance based on Criterion *2*, $K_{D}$, $K_{E}$, $K_{U}$ and $V_{Max,U}$ should decrease, whereas $D$and $V_{Max,C}$ should increase. However, the particular strategy of optimization with respect to $A$ and $K_{A}$ depends on Condition 1:

$$\left(\frac{K_{A}}{A}\right)^{2}>1-\frac{V_{Max,C}}{V_{Max,U}S}\left(\frac{K_{D}\text{ }K_{E}}{D}+K_{U}\right)$$

If Condition 1 is valid, then $A$ should increase and $K_{A}$ should decrease. On the other hand, if it is invalid, then $A$ should decrease and $K_{A}$ should increase. Furthermore, as was mentioned in the main text, if Condition 1 is valid, then optimization with respect to criteria *1* through *6* follows the same strategy.

**Table S1.** Evaluation of the Local Performance in Systemic Regime ***a***.

| ***Criterion*** | ***Capable***  ***of being fulfilled*** | ***Analytical Expression*** | ***Optimum value*** |
| --- | --- | --- | --- |
| *1* | + | $\frac{\frac{V_{Max,C}SD}{V_{Max,U}K_{E}K_{D}} \left(1+\frac{A}{K_{A}}\right)^{2}+\frac{A}{K_{A}}\left(5+4\frac{A}{K_{A}}\right)+\frac{K_{U}AD}{K_{E}K_{A}K_{D}}\left(4+3\frac{A}{K_{A}}\right)}{\frac{V_{Max,C}SD}{V_{Max,U}K_{E}K_{D}} \left(1+\frac{A}{K_{A}}\right)^{2}\left\vert1-\frac{\text{ }\frac{A}{K_{A}}\left(1+\frac{K_{U}D}{K_{E}K_{D}}\right)}{\frac{V_{Max,C}SD}{V_{Max,U}K_{E}K_{D}}\left(1+\frac{A}{K_{A}}\right)}\right\vert}$ | 1 |
| *2* | + | $\frac{\text{ }\frac{A}{K_{A}}\left(1+\frac{K_{U}D}{K_{E}K_{D}}\right)}{\text{ }\frac{V_{Max,C}SD}{V_{Max,U}K_{E}K_{D}}\left(1+\text{ }\frac{A}{K_{A}}\right)^{2}\left\vert1-\frac{\text{ }\frac{A}{K_{A}}\left(1+\frac{K_{U}D}{K_{E}K_{D}}\right)}{\frac{V_{Max,C}SD}{V_{Max,U}K_{E}K_{D}}\left(1+\text{ }\frac{A}{K_{A}}\right)}\right\vert}$ | 0 |
| *3* | + | $\frac{A\text{ }}{\text{ }\frac{V_{Max,C}SD}{V_{Max,U}K_{E}K_{D}}\left(K_{A}+A\right)\left\vert1-\frac{\text{ }\frac{A}{K_{A}}\left(1+\frac{K_{U}D}{K_{E}K_{D}}\right)}{\frac{V_{Max,C}SD}{V_{Max,U}K_{E}K_{D}}\left(1+\frac{A}{K_{A}}\right)}\right\vert}$ | 0 |
| *4* | + | $K_{A}+A$ | 1 |
| *5* | + | $A/\left(K_{A}+A\right)$ | 0 |
| *6* | + | $\left(V_{Max,C}^{Ref}(K_{E}K_{D}+K_{U}D)\right)/\left(V_{Max,C}DK_{C}^{Ref}\right)$ | 0 |
| *7* | + | $2+2K_{E}K_{D}/\left(K_{E}K_{D}+K_{U}D\right)$ | 2 |

It should be mentioned that there is one apparent conflict while trying to optimize performance with respect to all criteria. According to Criterion *7*, $K_{D}$ and $K_{E}$ should

decrease while $D$ and $K_{U}$ should increase. However, according to criteria *1*, *2* and *6* $K_{U}$ should decrease. This conflict is merely apparent because it can be readily resolved with appropriate values for $D$, $K_{D}$ or $K_{E}$ (for which there are no trade-offs).

## *Systemic Regime b*

In Table S2, we present the results from the analysis of local performance in Systemic Regime ***b***. Criteria *4* and *5* cannot be fulfilled in Systemic Regime ***b*** because the equation that governs the steady state does not depend on the concentration of

**Table S2.** Evaluation of the Local Performance in Systemic Regime ***b***.

| ***Criterion*** | ***Capable of being fulfilled*** | ***Analytical Expression*** | ***Optimum value*** | ***Parameters/variables***  ***that correlate*** | |
| --- | --- | --- | --- | --- | --- |
|  |  |  |  | **negatively** | **positively** |
| *1* | + | $3+\frac{K_{D}}{K_{D}+D}$ | 3 | $K_{D}$ | $D$ |
| *2* | + | 0 | 0 | ---^*^ | --- |
| *3* | + | $\frac{K_{D}}{K_{D}+D}$ | 0 | $K_{D}$ | $D$ |
| *4* | - | +∞ | NA^†^ | NA | NA |
| *5* | - | NA | NA | NA | NA |
| *6* | + | $\frac{K_{C}V_{Max,C}^{Ref}}{V_{Max,U}K_{C}^{Ref}}$ | 0 | $K_{C}$ | $V_{Max,U}$ |
| *7* | + | 2 | 2 | --- | --- |

^*^ The optimum value does not depend on the values of the parameters and independent variables.

^†^ NA – Not applicable

moiety-acceptor $A$ (see Table 1 of the manuscript). Hence, the supply of charged carrier will not respond to changes in the concentration of moiety-acceptor $A$ (Criterion *4*). It also becomes inconsequential to address how fluctuations in the values of the structural parameters and independent variables affect the sensitivity of $V_{C}$ to $A$ (Criterion *5*). Systemic Regime ***b*** has a fast response time (Criterion *6*), but not with respect to changes in $A$. Therefore, the importance of this responsiveness becomes questionable.

It is also worth mentioning that criteria *2* and *7* are capable of being fulfilled in Systemic Regime ***b*** and that this does not depend on any particular optimization strategy

## *Systemic Regime c*

In Table S3, we present the results from the analysis of local performance in Systemic Regime ***c***. As in Systemic Regime ***b***, criteria *4* and *5* cannot be fulfilled in Systemic Regime ***c***. In addition, even though Systemic Regime ***c*** can have a fast response time (Criterion *6*), it will not be with respect to changes in $A$. Therefore, as in Systemic Regime ***b***, the importance of this responsiveness becomes questionable.

All criteria are capable of being optimized with high values for $D$ and low values for $K_{D}$ and $K_{E}$ provided Condition 2 [Eqn. (S2)] is valid.

$$D^{2}>K_{D}^{2}K_{E}^{2}/\left(K_{C}K_{U}V_{Max,C}/V_{Max,U}+K_{U}^{2}\right)$$

However, if Condition 2 is invalid, then this optimization strategy will degrade the performance of Criterion *7*.

**Table S3.** Evaluation of the Local Performance in Systemic Regime ***c***.

| ***Criterion*** | ***Capable of being fulfilled*** | ***Analytical Expression*** | ***Optimum value*** | ***Parameters/variables***  ***that correlate*** | |
| --- | --- | --- | --- | --- | --- |
|  |  |  |  | **negatively** | **positively** |
| *1* | + | $\frac{6\text{+}\frac{D}{K_{D}}\left(5\frac{K_{U}}{K_{E}}\text{+}\frac{V_{Max,C}K_{C}}{V_{Max,U}K_{E}}\right)}{1\text{ + }\frac{D}{K_{D}}\left(\frac{K_{U}}{K_{E}}\text{+}\frac{V_{Max,C}K_{C}}{V_{Max,U}K_{E}}\right)}$ | 3 | $\begin{matrix} K_{D},\text{ }K_{E},\text{ }V_{Max,U},\text{ } \\ K_{U}^{*} \end{matrix}$ | $\begin{matrix} D,K_{C},V_{Max,C}, \\ K_{U}^{\dagger} \end{matrix}$ |
| *2* | + | 0 | 0 | ---^‡^ | --- |
| *3* | + | $1/\left(1\text{ +}\frac{D}{K_{D}}\left(\frac{K_{U}}{K_{E}}+\frac{V_{Max,C}K_{C}}{V_{Max,U}K_{E}}\right)\right)$ | 0 | $K_{D},\text{ }K_{E},\text{ }V_{Max,U}$ | $\begin{matrix} D,\text{ }K_{C},\text{ }V_{Max,C},\text{ } \\ K_{U} \end{matrix}$ |
| *4* | - | +∞ | NA^§^ | NA | NA |
| *5* | - | NA | NA | NA | NA |
| *6* | + | $\frac{\frac{K_{C}}{K_{C}^{Ref}}\left(1+\frac{K_{U}D}{K_{E}K_{D}}\right)}{\frac{V_{Max,U}}{V_{Max,C}^{Ref}} \left(1+\frac{K_{U}D}{K_{E}K_{D}}\right)+\frac{V_{Max,C}K_{C}D}{V_{Max,C}^{Ref}K_{E}K_{D}} }$ | 0 | $\begin{matrix} K_{C},K_{D},K_{E}, \\ K_{U} \end{matrix}$ | $D,\text{ }V_{Max,C},\text{ }V_{Max,U}$ |
| *7* | + | $2+2\frac{\frac{V_{Max,C}K_{C}D}{V_{Max,U}K_{E}K_{D}}}{\left(1+\frac{K_{U}D}{K_{E}K_{D}}\right)\left(1+\frac{D}{K_{D}}\left(\frac{K_{U}}{K_{E}}+\frac{V_{Max,C}K_{C}}{V_{Max,U}K_{E}}\right)\right)}$ | 2 |  |  |

^*^ Condition 3 ($D>K_{D}K_{E}V_{Max,U}/4K_{C}V_{Max,C}$) true; ^†^ Condition 3 false; ^‡^ The optimum value does not depend on the values of the parameters and independent variables; ^§^ NA – Not applicable; ^¶^ Condition 2 ($D^{2}>K_{D}^{2}K_{E}^{2}/\left(K_{C}K_{U}V_{Max,C}/V_{Max,U}+K_{U}^{2}\right)$) true; ^||^ Condition 2 false.

Furthermore, several other trade-offs exist:

• In order to optimize local performance according to criteria *1* and *3*, $V_{Max,U}$ should tend to low values and $K_{C}$ should tend to high values. However, the opposite is true for optimizing criteria *6* and *7*

• To improve criteria *1*, *3* and *6*, $V_{Max,C}$ should tend to high values, while to improve Criterion *7*, $V_{Max,C}$ should tend to low values.

Finally, the optimum value of Criterion *1* in Systemic Regime ***c*** is 1, whereas that in Systemic Regime ***b*** is 3. Therefore, since Systemic Regimes ***b*** and ***c*** share the same optimum values for the remaining criteria, we conclude that overall local performance in Systemic Regime ***c*** is better than that in Systemic Regime ***b***.
