# Supplementary material for: Quantifying Global Tolerance of Biochemical Systems: Design Implications for Moiety-Transfer Cycles
Source: PLoS Comput Biol. 2009 Mar 20;5(3):e1000319. doi: 10.1371/journal.pcbi.1000319 (PMC2650413; doi:10.1371/journal.pcbi.1000319)
Supplement: Text S3 — Tolerance expressions (0.72 MB DOC) [file pcbi.1000319.s003.doc]

**Supporting Text S3**

## *Tolerance Expressions*

**Table S4**. Expressions for the tolerances of variables and parameters that characterize systems operating in Systemic Regime ***a***. Tolerances and conditions relative to each of the two boundaries are derived from the piecewise power-law representation. The tolerance is infinite when the corresponding condition is not satisfied. The superscript Op signifies the normal operating value for a variable or parameter.
