# Supplementary material for: Quantifying Global Tolerance of Biochemical Systems: Design Implications for Moiety-Transfer Cycles
Source: PLoS Comput Biol. 2009 Mar 20;5(3):e1000319. doi: 10.1371/journal.pcbi.1000319 (PMC2650413; doi:10.1371/journal.pcbi.1000319)
Supplement: Text S4 — NADPH redox cycle in human erythrocytes (0.07 MB DOC) [file pcbi.1000319.s004.doc]

**Supporting Text S4**

## *NADPH redox cycle in human erythrocytes*

**Table S5**. Numerical values of the parameters and concentration variables for the two enzymes of the NADPH redox cycle in human erythrocytes.

| **Glucose 6-Phosphate Dehydrogenase (G6PD) (Charging Enzyme)** | | **Glutathione Reductase (GSR)**  **(Uncharging Enzyme)** | |
| --- | --- | --- | --- |
| **Parameter** | **Value** | **Parameter** | **Value** |
| $V_{Max,C}$ | 130 μM.s^-1^ ^*,†^ | $V_{Max,U}$ | 49 μM.s^-1^ ^*^ |
| $D$ | 39 μM ^‡^ | $A$ | 0.16 μM ^║^ |
| $K_{U}$ | 18 μM ^§, ¶^ | $K_{C}$ | 8.5 μM^**^ |
| $K_{D}$ | 38 μM ^§^ | $K_{A}$ | 65 μM ^**^ |
| $K_{E}$ | 7.9 μM ^§^ | $S=C+U$ | 2.7 μM ^║^ |

^*^ ref [1]; ^‡^ ref [2]; ^§^ ref [3]; ^║^ ref [4];^**^ ref [5]

^†^ For the NADPH redox cycle, we are considering that the maximum velocity of the charging enzyme is twice the maximum velocity of glucose 6-phosphate dehydrogenase. For further details see [4].

^¶^ The $K_{U}$ we are using takes into account product inhibition as well as the inhibition by 2,3-diphosphoglycerate. For further details see [4].

# *References*

1. Thorburn DR, Kuchel PW (1985) Regulation of the human-erythrocyte hexose-monophosphate shunt under conditions of oxidative stress. A study using NMR spectroscopy, a kinetic isotope effect, a reconstituted system and computer simulation. Eur J Biochem 150: 371-386.

2. Beutler E (1984) Red cell metabolism: a manual of biochemical methods. New York: Grune and Stratton.

3. Kirkman HN, Wilson WG, Clemons EH (1980) Regulation of glucose-6-phosphate dehydrogenase. I. Intact red cells. J Lab Clin Med 95: 877-887.

4. Salvador A, Savageau MA (2003) Quantitative evolutionary design of glucose 6-phosphate dehydrogenase expression in human erythrocytes. Proc Natl Acad Sci U S A 100: 14463-14468.

5. Worthington DJ, Rosemeyer MA (1976) Glutathione reductase from human erythrocytes. Catalytic properties and aggregation. Eur J Biochem 67: 231-238.
